# Virulence of current German PEDV strains in suckling pigs and investigation of protective effects of maternally derived antibodies

**DOI:** 10.1038/s41598-017-11160-w

**Published:** 2017-09-07

**Authors:** S. Leidenberger, Ch. Schröder, L. Zani, A. Auste, M. Pinette, A. Ambagala, V. Nikolin, H. de Smit, M. Beer, S. Blome

**Affiliations:** 1 Institute of Diagnostic Virology, Friedrich-Loeffler-Institut, Suedufer 10, Greifswald, Insel Riems 17493 Germany; 2 Canadian Food inspection Agency, National Centre for Foreign Animal Disease, Winnipeg, R3E 3M4 Canada; 3 Boehringer Ingelheim Veterinary Research Center, Hannover, 30559 Germany

## Abstract

Porcine epidemic diarrhea (PED) has caused tremendous losses to the United States pig industry since 2013. From 2014, outbreaks were also reported from Central Europe. To characterize the Central European PEDV strains regarding their virulence in suckling piglets, and to assess the protective effect of maternally derived antibodies (MDA), four trial groups were randomly assigned, each consisting of two pregnant sows and their litter. To induce MDA in a subset of piglets, two sows received a cell culture-adapted PEDV strain, and another two sows were inoculated with field material from German PED outbreaks. Four sows stayed naïve. Subsequently, all piglets were inoculated with the corresponding PEDV strains at an age of 3 to 6 days, and virus shedding, clinical signs and occurrence of specific antibodies were assessed. Piglets without MDA showed a morbidity of 100% and low lethality, while almost all MDA-positive piglets stayed clinically healthy and showed considerably lower virus shedding. Taken together, the Central European PEDV strains showed rather low virulence under experimental conditions, and pre-inoculation of sows led to a solid protection of their offspring. The latter is the prerequisite for a sow vaccination concept that could help to prevent PED induced losses in the piglet sector.

## Introduction

Porcine epidemic diarrhea (PED) is a highly contagious enteric disease of swine, affecting pigs of all ages. Severe clinical signs and high mortality rates are especially observed in suckling piglets and severity of disease decreases with age. However, strain virulence and management conditions also impact on the clinical disease and outcome^1–3^.

The disease is mainly characterized by watery diarrhea and vomiting which leads to dehydration and deteriorating constitution. The causative agent is PED virus (PEDV), an enveloped, single-stranded, positive-sense RNA virus of the genus *Alphacoronavirus* within the *Coronaviridae* virus family of the order *Nidovirales* ^4^.

Porcine epidemic diarrhea first emerged in 1971 in England termed Epidemic Diarrhea (ED)^5, 6^. These early outbreaks were characterized by severe clinical signs and mortality rates of up to 100% in young fattening pigs. In the beginning, no cases were reported from suckling pigs. Later on, pigs of all ages showed similar disease in different European countries, and over the following years, PED was reported from Belgium, the United Kingdom (UK), The Netherlands, Germany, Hungary, Bulgaria, France, Switzerland, and Spain^7–9^. For unknown reasons, the occurrence of PED in Europe decreased markedly after the 1980’s evidenced by lacking reports of further outbreaks and a very low seroprevalence in the nineties and the following years^10, 11^. There were only sporadic outbreaks reported associated with low mortality, e.g. in The Netherlands, Hungary and the UK^12–14^. In contrast, PEDV caused outbreaks of higher economic impact in Asia^15, 16^. Still, endemic infections are reported from Asia, and the impact on the productivity of industrialized pig farms remains high^17, 18^. Since 2005, PED cases were again sporadically reported from Europe, i.e. Italy, but without much leverage on the pig industry^19^.

In 2011 and 2012, new, highly virulent PEDV variants were reported from China that caused tremendous losses, especially in the piglet sector^20, 21^. In the following, starting in April 2013, PED occurred for the first time in the United States (US) and rapidly spread through nearly the whole country. High mortality rates of up to 95% were reported among suckling pigs, which led to unimaginable economic losses of whole piglet batches. The exact origin and introduction route of these highly virulent PEDV strains could not be finally elucidated^22, 23^. Phylogenetic analyses showed that the causative strains cluster together with the above mentioned Chinese isolates from 2011 and 2012^24–26^. With the disease threatening to spread, European farmers and veterinarians were alerted and asked for data on PED prevalence and possible impact. Consequently, surveillance in Europe was reinforced and PED cases were indeed reported from Central Europe i.e. Germany^27^, the Ukraine^28^, France^29^, Austria^30^, Portugal^31^ and Belgium^32^. In Germany, cases were reported starting from May 2014 especially in the South-Western and North-Western part^25, 27, 33^. In general, the impact on the pig industry remained low. Retrospective analyses demonstrated that a considerable seroprevalence was present only in Italy^34^. Thus, a recent re-introduction is most likely.

Based on partial and full-genome sequences, strains from recent US outbreaks are clearly distinct from the original European PEDV strains^25^, and two different virus types were found. On the one hand there are so called “original US PEDV”, which are commonly referred to as NON-INDEL strains (meaning no insertions and deletions in the open reading frame (ORF) encoding for the spike protein), and on the other hand so called “S-INDEL” variants that show insertions and deletions in spike protein encoding region^35^. The latter were supposed to cause milder disease course with low mortality. With exception of PEDV strains from Ukraine^28^, the current European strains were identified as S-INDEL strains closely related to OH851 from the U.S.^25^. However, field virulence and mortality rates differed between European countries with low overall impact^25, 28, 29, 33, 36^. In Germany, mortality ranged from almost zero to more than 70% in suckling pigs with almost complete genetic identity of the causative PEDV strains^25, 33^. Here we report about our studies of both the virulence of recent S-INDEL PEDV strains from Central Europe under controlled laboratory conditions, and the protective effect of maternally derived antibodies (MDA). Up to now, PED control is mainly achieved by strict veterinary hygiene and biosecurity, and vaccination is not practiced outside of Asia and - more recently - America. However, taking into account the current threats, vaccination strategies are revisited. To protect the most susceptible host, the suckling pig, mainly a sow vaccination concept seems reasonable. A prerequisite would be a solid protection through maternally derived antibodies (MDA).

This aspect was investigated in our study for two Central European PEDV strains. The trial design involved four groups, each consisting of two pregnant sows and their litter. To induce MDA in a subset of piglets, two sows received a cell culture-adapted PEDV strain (A1), and another two sows were inoculated with field material from German PED outbreaks (B1) four weeks before farrowing. Four sows stayed naïve (groups A2 and B2). Subsequently, all piglets were inoculated with the corresponding PEDV strains at an age of 3 to 6 days, and virus shedding, clinical signs and occurrence of specific antibodies were assessed. It was demonstrated that MDA have a strong protective effect and thus, further studies into vaccination concepts are warranted.

## Results

### Clinical and pathological observations

#### Clinical signs upon inoculation of sows

Two sows, which were inoculated with a cell culture-adapted PEDV strain (PEDV EU, group A1) showed slight depression over a period of four days, accompanied by reduced feed intake.

In contrast, the two sows of group B1 (inoculated with German field PEDV, DE) showed diarrhea and vomiting upon inoculation. Moreover, both sows were anorectic for one day. Thereafter, one of the sows (ear tag 6150) was still showing slight depression over four days whereas the other was free of clinical signs again.

All four sows of groups A2 and B2, which stayed naïve prior to farrowing, remained healthy during the pre-farrowing time.

#### Clinical signs upon inoculation of piglets

Sows which were naïve before farrowing and got infected via their inoculated piglets during the trial, showed more severe clinical signs according to our standardized score system for sows (Table 1) than sows, which were directly inoculated four weeks before farrowing (see above). Almost complete anorexia, watery diarrhea, and decrease in milk production was observed from 60 h after the inoculation of the piglets in sows of groups A2 and B2 (see Supplementary Fig. F1). The lack of milk production led to a more severe situation for their unprotected infected piglets (malnutrition).

**Table 1:** Clinical score system for sows in PED infection studies. The humane endpoint was defined as a cumulative score >6. Moreover, euthanasia was carried out after abortion or other signs indicative for inacceptable suffering.

Score	General behavior	Feed intake/suckling	Gastrointestinal signs
0	No abnormalities	No abnormalities	Physiological feces
1	Mild depression, reluctance to move	Reduced interest in feed	Pasty feces
2	Reduced general condition, extended resting, indication of decreasing milk production	Hardly interested in feed without clear feed intake	Watery feces, reddened anal region, vomiting
3	Strong depression, almost entirely resting, decreased milk production, abortion	Total anorexia	Watery feces with blood or fibrin added, highly reddened anal region, vomiting

Sows, which were inoculated before farrowing (sows of groups A1 and B1), did not show any clinical signs indicative for PED upon challenge of their piglets. One sow of group A1 died from septicaemia two days after farrowing (necropsy revealed severe peritonitis after uterine rupture and intestinal invagination). The respective piglets (which had all taken colostrum) were reared by the remaining sow of group A1.

**Figure 1:**
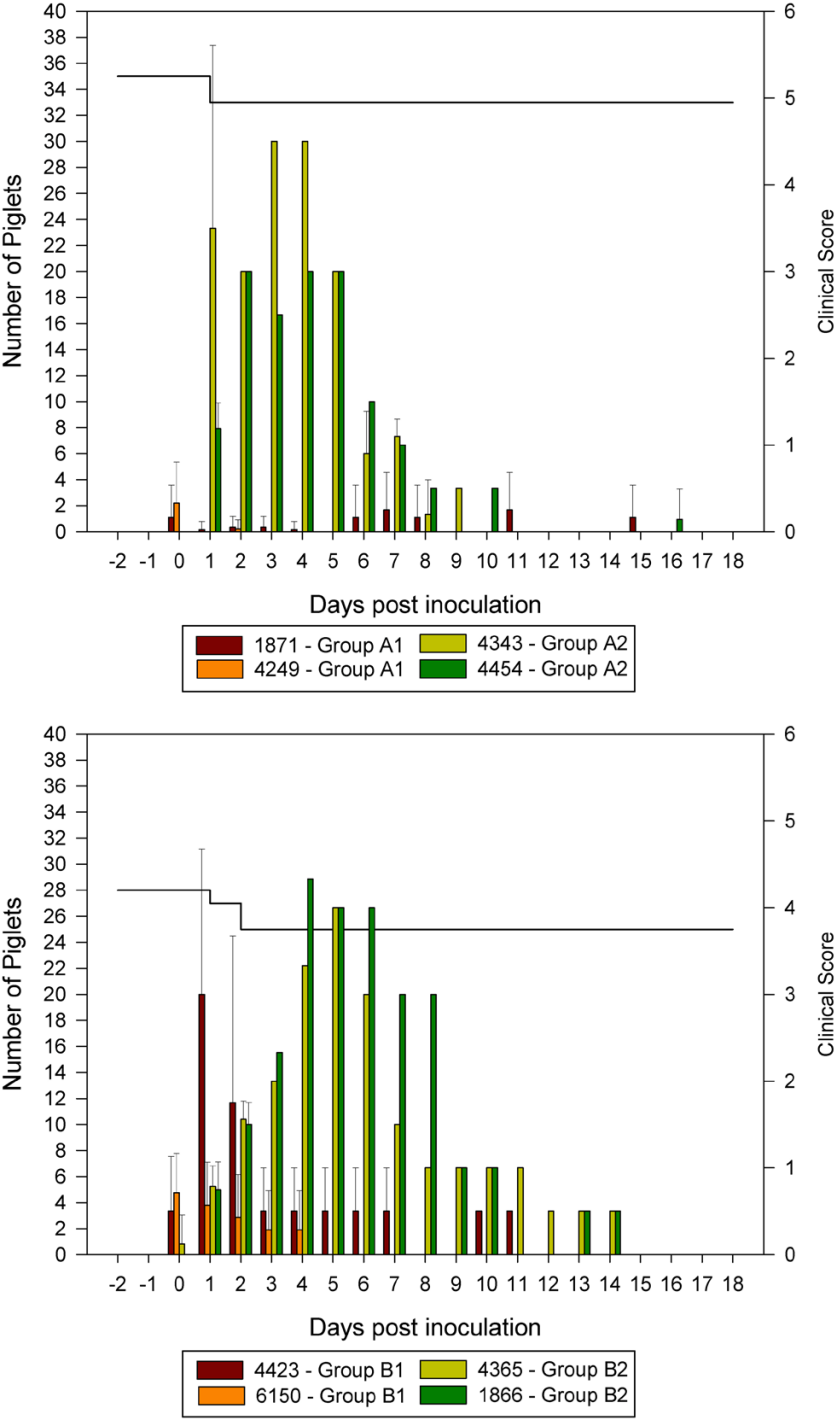
Cumulative clinical scores and piglet survival shown per litter per sow: The bars indicate the clinical scores of each litter per sow and the black line across the top of the graph represents the number of surviving piglets over time; upper chart: animals inoculated with cell culture adapted PEDV-strain (Group A1 (sows 1871 and 4249) and A2 (sows 4343 and 4454)), lower chart: animals inoculated with German field material (Group B1 (sows 4423 and 6150) and B2 (sows 4365 and 1866)), standard deviation is shown in error bars.

As can be seen in Fig. 1, none of the MDA-positive piglets showed any severe clinical signs indicative for PED upon challenge inoculation. Very mild signs were discontinuously observed in some animals (pasty feces). One litter (group B1, sow 4423) showed general weakness and remittent shivering leading to clinical scores prior to inoculation. A viral genesis could not be detected by routine PCRs (Porcine enteroviruses (PEV), Suid alphaherpesvirus-1, Classical and African swine fever virus, Atypical porcine pestivirus).

Piglets without MDA and inoculated with the cell culture-adapted virus (PEDV EU, group A2) started vomiting 24 hours post inoculation (hpi), and showed diarrhea from 36 hpi. The clinical signs remained over 6 to 7 days with diarrhea as the leading sign (see Fig. 1, upper graph). MDA-negative piglets challenged with the German field material (DE), started to show signs indicative of PED at 24 hpi, also beginning with vomiting followed by diarrhea. Clinical signs persisted over 10 to 11 days (Fig. 1, lower graph). As the respective sows showed an almost complete cessation of milk production, piglets showed signs of malnutrition. Two piglets had to be euthanized with an endpoint clinical score because of dehydration and general weakness. Necropsy of the euthanized piglets showed gas-filled intestines. All other piglets were without specific results indicative for PED at the time point of necropsy (end of the trial).

Clinical score values differed significantly between groups with and without MDA from 2 to 10 days post inoculation (dpi). In addition, a statistically significant difference was observed at 1 dpi between groups A1 and A2, and 12 to 14 dpi between groups B1 and B2. The above mentioned condition in one litter of group B1 led to a significant difference at the day of challenge that was however not PED related.

### Shedding of virus

#### PEDV shedding among sows

Sows infected with cell culture-adapted PEDV (group A1) shed virus from 4 or 5 days post inoculation (dpi) for 7 to 10 days, while the group inoculated with the field virus (B1) was PCR-positive from 4 dpi for 9 to 10 days. All naïve sows were negative prior to the inoculation of piglets.

### PEDV shedding of piglets

**Figure 2:**
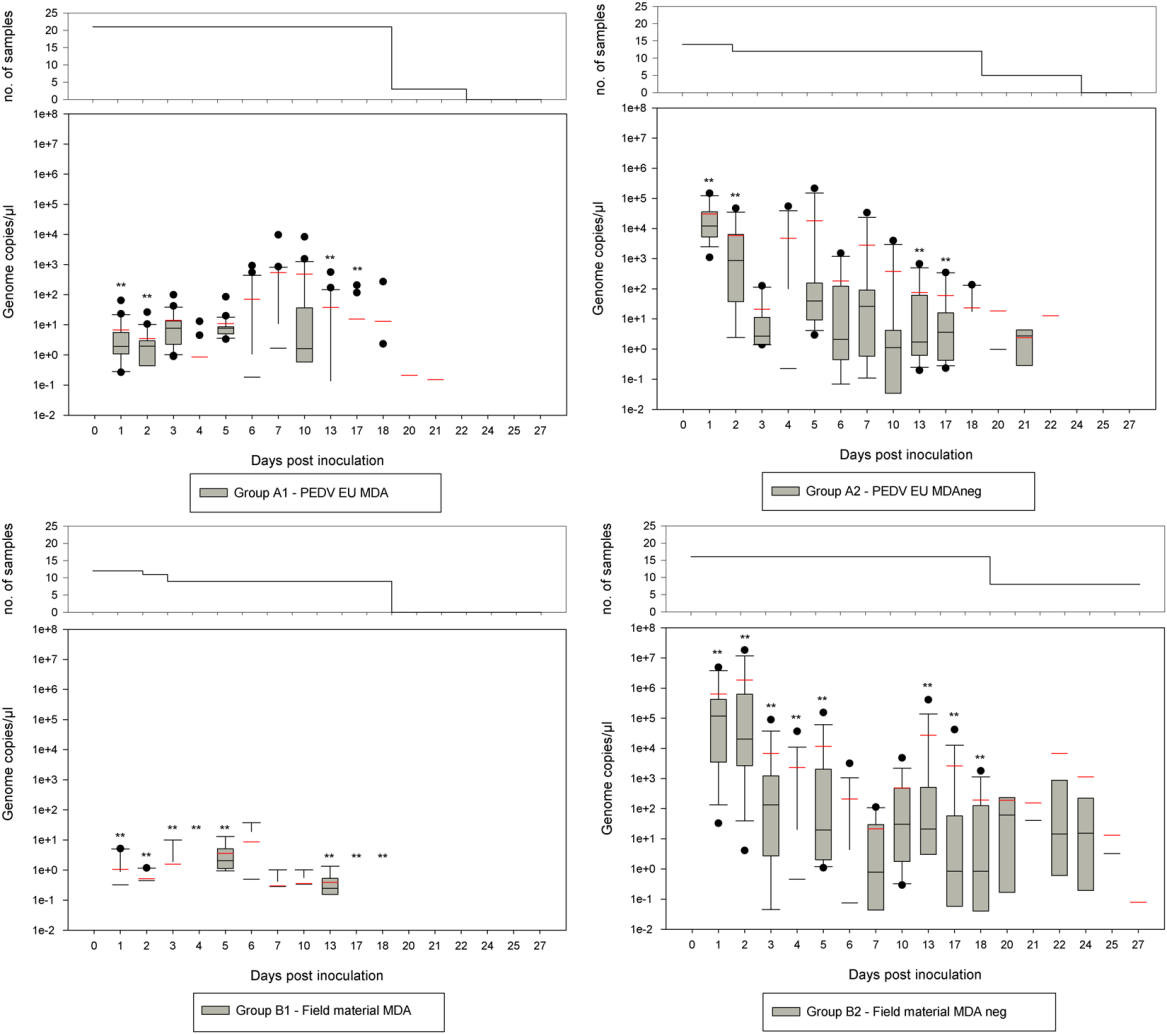
PEDV-shedding detected via RT-qPCR: The graph shows the amount of genome copies per group using a logarithmic scale with 1e + 8 meaning 10^8^; red lines mark the mean (average) of each box plot, black lines mark the median of each box plot; upper chart: Group A1 and A2 (cell culture isolate; PEDV EU), lower chart: B1 and B2 (German field material, DE); statistically significant differences between groups with and without maternal derived antibodies (compared by day) are marked with **(p-values between 0.001 and 0.034). To indicate the number of samples included in each boxplot, the survival curve is given above the respective figure part.

Detection of PEDV genome occurred in all groups but to a much lesser extent in the MDA-positive animals (see Fig. 2, PEDV EU in the upper graph, field material DE in the lower). In general, the amount of detectable PEDV genome was influenced by the faecal load on the swab. However, as this phenomenon occurred in all groups, it was regarded as systematic error. All below mentioned genome copy numbers refer to 1 µl RNA extracted from 100 µl faecal suspension (all swabs were submerged in 1 ml medium, irrespective of their load).

Piglets of groups A1 and A2 showed viral RNA in rectal swabs beginning 1 dpi until 20 dpi, and the genome load was markedly lower in piglets of group A1 with MDA. Significant differences were found among groups A1 and A2 on 1, 2, 13, and 17 dpi with higher genome loads in the animals without MDA (A2). On the other days, the genome loads were still higher in group A2, but not significantly different. It was quite the same in group B. In this case, genome loads were significantly higher on 1 to 5 dpi and 13 to 18 dpi in piglets without MDA (B2).

In more detail, all animals of group A2 were found positive at 1 dpi with genome loads between 1100 and 147000 genome copies per µl, whereas only low virus shedding could be detected in group A1 with genome copy numbers ranging from 0.2 to 64 per µl (see Fig. 2, upper chart). At 2 dpi, a comparable picture was seen at lower level. However, single results varied considerably (genome copies ranging from 0 to 47000 in group A2 and 0 to 26 in group A1). Only low copy numbers were detected for all animals at 3 dpi (no significant differences). Up to 7 dpi, mainly low genome loads were detected with single animals showing considerable shedding (up to 213500 copies per µl for a MDA negative piglet at 5 dpi). At 7 dpi all but one piglets of group A2 still showed genome loads of 0.4 to 33000 genome copies per µl. Animals of group A1 showed generally lower genome loads with 0.2 to 850 genome copies per µl. Only one piglet of this groups shed higher genome loads with 9600 genome copies per µl. Thereafter, shedding decreased, with faster decline in the MDA positive groups (see Fig. 2, upper graph).

Compared to group A2, MDA negative animals of group B2 showed higher initial virus shedding (up to 18000000 copies per µl) at 1 and 2 dpi. Only a few animals of the MDA positive group were detected positive for a few days with genome loads ranging from 0.15 and 37 genome copies. From 3 dpi, virus shedding decreased gradually with scattered positive findings and high variability. From 17 dpi, all MDA positive animals (B1) were negative while shedding still occurred in MDA negative animals of group B2 (see Fig. 2, lower graph).

### Detection of other relevant pathogens

The inoculum of the sows was tested negative for transmissible gastroenteritis virus and porcine delta coronavirus. Positive results were obtained for porcine circovirus 2, rotavirus A, and porcine enteroviruses (PEV). The piglet inoculum was tested positive for PEV genome (cq value: 30) and showed negative results for all other pathogens. However, no PEV-shedding could be detected in piglets, which showed signs that might have been indicative for PEV infection (group B2; litter of sows 4423 and 6150).The cell culture adapted virus (PEDV EU, groups A1 and A2) was free of all tested pathogens.

### Antibody detection in serum and colostrum

Prior to the study, sows were tested for PEDV-specific antibodies by three commercial indirect IgG isotype antibody ELISA. All but one animal showed clear negative results. The serum sample of the remaining sow which had shown inconclusive results was retested by indirect immunofluorescence with clear negative results.

**Figure 3:**
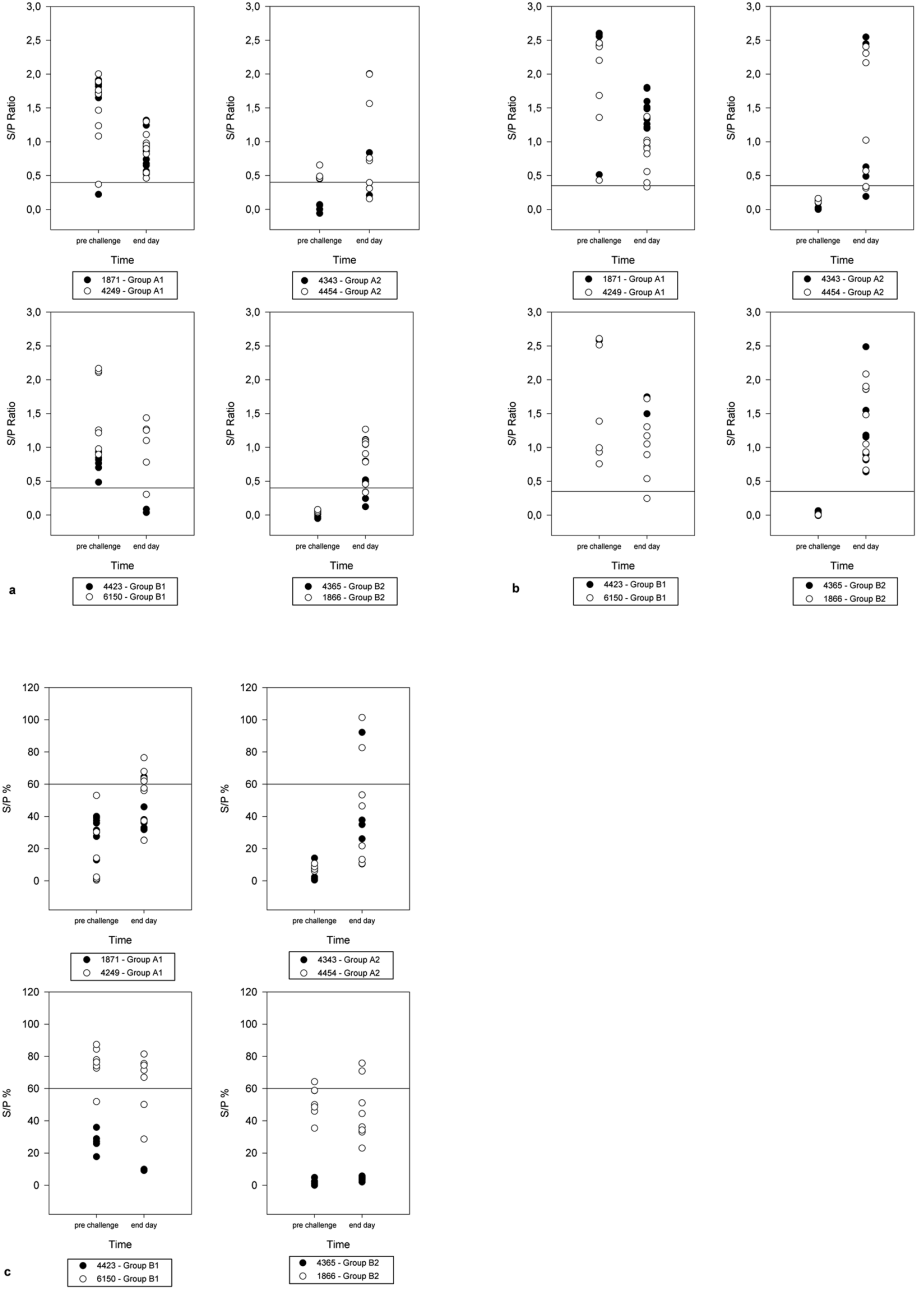
IgG detection in serum samples of piglets prior to challenge and at day of death using commercial indirect ELISA kits: Comparison of three commercial ELISA assays for PEDV specific IgG antibodies, results are shown as sample-to-positive ratio for each individual piglet, the horizontal line marks ELISA cut-off; (**a**) BIOVET; (**b**) INGENASA; (**c**) IDVet; upper graphs: PEDV cell culture isolate (PEDV EU): upper left: Group A1, upper right: Group A2; lower graphs: German field material (DE): lower left: Group B1, lower right: Group B2.

All sows, which were inoculated with PEDV prior to farrowing (groups A1 and B1), showed IgG isotype antibodies in blood and colostrum samples at the day of farrowing using the ELISA assays mentioned above. In general, the used ELISA kits seemed to vary regarding specificity and sensitivity. Nevertheless, the overall tendency of antibody levels in the different groups was comparable (see Fig. 3a–c).

Colostrum samples were also tested positive for PEDV S protein specific IgA isotype antibodies using an in-house ELISA. In those pre-inoculated animals, antibody levels in blood decreased till the end of the trial. Sows in groups A2 and B2 had no detectable PEDV specific IgG isotype antibodies in blood or colostrum at the day of farrowing but developed rising levels till the end of the trial once they got exposed to PEDV from their offspring.

Blood samples of all but two piglets received from pre-inoculated sows (both in group A1) were shown to contain PEDV antibodies prior to inoculation (see Fig. 3a–c, upper right chart for PEDV EU and lower right chart for DE). At the end of the trial 72.0% (B1/B2) to 81.8% (A1/A2) of the piglets and all of the sera from the sows were positive in the IgG-ELISA (see Fig. 3a–c). All milk samples collected at the day of slaughter or death were negative for PEDV-specific IgG, but showed varying amounts of IgA isotype antibodies. This was also found in samples of sows which were naïve prior to farrowing (groups A2 and B2) (see Supplementary Fig. F2).

As can be seen in Fig. 3a–c, a clear increase of serum antibodies was observed over the course of the trial in MDA negative piglets of groups A2 and B2, whereas antibody levels markedly decreased in the MDA positive groups A1 and B1.

### Bacteriology

The fecal swabs taken at 0 dpi and 2 dpi did not show any growths of pathogenic bacteria.

The bacterial flora was similar in the samples at 0 and 2 dpi.

## Discussion

Following the disastrous PED outbreaks in the US, re-introduction was also confirmed in Central Europe^25, 27, 29–32, 34^. With the exception of the highly virulent strains occurring in Ukraine^28^, all recent European PEDV strains were so-called S-INDEL variants with an expected lower virulence than the US-type NON-INDEL PEDV variants. Overall, the PED impact on pig industry seemed to be low, but sporadic cases with high mortality and long persistence of the disease in continuously producing enterprises was observed^37^.

Follow-up investigations showed that the reported virulence of the German PEDV strains varied considerably among breeding herds (suckling pigs) while only mild diarrhoea was observed in fatteners. Interestingly, the causative strains were highly identical, even on the full-genome level^33^. Detailed molecular analyses did not reveal any significant impact of viral and bacterial co-infections or viral variants^38^.

In order to assess strain-related virulence and disease dynamics among suckling pigs in the presence and absence of maternally derived antibodies, the presented study was carried out under controlled laboratory conditions. In a nutshell, the PEDV strains obtained from recent German outbreaks (both cell culture and field material) showed indeed a low virulence with only two MDA-negative piglets that had to be euthanized at the humane endpoint. All remaining piglets recovered completely and were able to compensate the weight loss by the weaning age. However, piglets with MDA were almost completely protected from clinical disease. In direct comparison, the field material induced more severe signs in naïve piglets than the cell culture isolate. Possible reasons include attenuation by cell culture adaptation but also differences in inoculation dose and secondary pathogens. Regarding the inoculation dose, it can be stated that the genome load was higher in the cell culture material than in the field material. Provided that this gives at least an indication for the virus titer, we can assume that the virus titer of the field material was at least not considerably higher than that of the cell culture material. Testing of the field materials for secondary pathogens showed low rotavirus A and enterovirus loads. The secondary materials used for the inoculation of the piglets in groups B1 and B2 contained PEDV and low PEV genome loads. The latter was however not found in any of the piglets.

Despite the observed differences, 100% morbidity and long-term shedding of viral RNA was still observed for both variants in MDA-negative piglets.

In contrast to the study with a cell culture adapted US-type PEDV, published by Poonsuk *et al*. in 2016^39^, all sows that were naïve prior to farrowing showed diarrhea after challenge of their piglets. Disease progression in these sows was worse than the disease course in the sows following direct inoculation prior to farrowing (groups A1 and B1). Diarrhea and depression even led to a severe decrease of milk production and thus inadequate nutrition of piglets. This underlines the importance of the sow’s health status for the fate of PED-diseased piglets. The increased severity could probably be explained by the more fragile immune system during the farrowing and lactating period, but also with the higher virus loads in the stable and ongoing contact to PEDV shed by the infected piglets^39^.

Shedding of viral RNA in both groups was detected over three weeks and beyond, which is in line with former studies^30, 33, 40^ and field observations. The variability of individual results was probably due to the different content of fecal material on the swab. However, also the desquamation of intestinal cells and hereby the amount of virus at rectal swabs might vary. In order to gain individual samples, pooled faecal samples were not considered appropriate. The long-term shedding of virus could be a most important issue for disease control and elimination. With shedding over such a long time, it is possible that clinically healthy but still shedding piglets are sold and brought to another holding for subsequent production steps where they pose a risk for naïve stable/pen mates. The risk of disease transmission by those piglets would however need further investigation.

Most importantly, our study clearly shows that maternally derived immunity against PEDV is able to protect piglets in the most vulnerable phase after birth. This is prerequisite for a sow vaccination strategy which should be explored in light of the tremendous impact of PED in the US and also farms in the Ukraine. With neonatal piglets being born agammaglobulinemic and possessing limited, undeveloped lymphoid tissues and no effector and memory T-lymphocytes^41^, it is absolutely necessary that they receive maternal cell-mediated and humoral immune components through the ingestion of colostrum and milk^42^. It is already shown for TGEV that natural oral infection of sows leads to a more effective maternal derived lactogenic protection for suckling pigs than systemic immunization^43, 44^. This effect is mainly based on a larger amount of secretory IgA in colostrum and milk because of stimulation of the gut-mammary-axis of the sow. Systemic immunization usually induces high levels of IgG in both the blood and colostrum, but IgA titers in blood might stay on a low level offering no reliable lactogenic protection against infection of piglets^45^. This is in line with the results of the provided study, in which high amounts of IgG could be detected in colostrum but not in milk samples, whereas IgA could be found in both – colostrum of pre-infected sows and milk of all sows. Thus, also the sows that stayed naïve prior to farrowing seroconverted upon challenge of their piglets and low levels of IgA could be detected in milk samples at the end of the trial. When revisiting PED vaccination, this route-dependent issue should be kept in mind as it could explain the unsatisfactory protection against virus shedding and morbidity of piglets from sows that were only parenterally immunized using different types of vaccines^46^.

Therefore, a sow vaccination concept against PEDV should probably prefer oral or other mucosal immunization routes for proper protection of suckling pigs if possible^9, 47, 48^.

Regarding the duration of immunity in both sows and piglets, we could confirm that the antibodies are rather short-lived. Sows pre-exposed prior to farrowing showed already decreasing levels of IgG at the end of the trial (with ELISA results still being positive). With a high antibody titer in the blood being a requirement for solid and protective antibody titers – especially secretory IgA - in colostrum and milk, it seems that sows would have to be re-vaccinated/re-infected prior to each farrowing to ensure piglet protection. Moreover, immunization is needed close to the farrowing date. From literature it is known that antibody levels of IgG and IgA in serum of primiparous and multiparous sows can remain stable up to 6 months post field infection as measured by high titers in ELISA and virus neutralization assay^49^. The respective sows were obtained from commercial breeding farms that had reported previous PED outbreaks. Thus, booster/reinfection seems to play a role for the duration of immunity. With regard to piglets, field studies observed antibody levels in piglets that lasted up to 100 days^49, 50^. For the piglets/young weaners, the source of antibodies clearly influences the duration of detection. While a decay of MDA is visible already after a few weeks, antibodies induced by active immunization or infection might last much longer. In our study, MDA-positive animals had only low antibody levels by the end of the trial (at the age of weaning). In these animals, protection in later stages of pig production would have been questionable and this could explain field observations that showed reinfection of finishing pigs that had been exposed to PEDV as suckling pigs. These aspects also need consideration when designing a vaccination concept.

Another important fact is the reliability of commercial ELISA assays. In this study three commercial ELISA assays were compared and variations in specificity and sensitivity were found as already shown in other studies^51^. Therefore, the combined use of different diagnostic tools to investigate the serological status of an animal should be taken into account.

In conclusion, the tested PEDV strains were of moderate to low virulence and natural infection of sows during gestation led to an effective lactogenic immunity in piglets. This is a promising outcome when vaccination concepts are discussed.

As a spin-off of our trial, a simple and standardized score system was implemented that worked well even with limited clinical signs. The presented score system (see Table 2) is less detailed than others that were previously described^52^. Nevertheless, it seems sufficiently reliable and will be used for subsequent immunization-challenge experiments.

**Table 2:** Clinical score system for piglets in PED infection studies. The humane endpoint was defined as a cumulative score >6. Moreover, euthanasia was carried out if one of the following criteria occurred: body temperature <38.5 °C, lateral recumbency, severe dehydration, complete suspension of feed intake.

Score	General behavior	Feed intake/suckling	Gastrointestinal signs
0	Agile, attentive, no abnormalities	Greedy suckling, good filled stomach, intake of piglet feed	Physiological feces
1	Mild depression	Slow suckling, hardly interested in piglet feed	Pasty feces, vomiting
2	Depression, isolaton from group, vocalisation (moaning)	reluctant feed intake, hardly interested in suckling/piglet feed, sunken flanks	Watery feces, reddened anal region, vomiting
3	Reluctance to stand, signs of severe dehydration, low body temperature	Total anorexia, decreasing of milk production of sow	Watery feces with blood or fibrin added, highly reddened anal region, vomiting

## Material and Methods

### Study Design

In total, eight sows and their offspring were used in this study. The multiparous sows were purchased in late pregnancy from a commercial breeding farm with a high veterinary hygiene standard (Bundes Hybrid Zucht Programm, BHZP, Dahlenburg-Ellringen, Germany). They were transported to the high containment facilities at the Friedrich-Loeffler-Institute Greifswald, Insel Riems, Germany, five weeks before farrowing. According to EU legislation, the animals were kept in open pens in sets of two before they were moved to commercial farrowing pens one week before farrowing. Two farrowing pens were integrated in one stable room so that each experimental group could be kept together. All animals had access to water *ad libitum* and were fed with commercial feed for breeding sows and after farrowing for lactating sows. All applicable animal welfare regulations, including EU-Directive 2010/63/EC and institutional guidelines, were taken into consideration. The animal experiment was approved by the competent authority, Landesamt für Landwirtschaft, Lebensmittelsicherheit und Fischerei Mecklenburg-Vorpommern (LALLF MV), under reference number LALLF 7221.3-1-059/16.

All animals were tested prior to arrival with negative PCR-results for PEDV and antibodies against PEDV. After arrival, the animals were randomly assigned to four treatment groups. Two sows (ear tags 1871 and 4249) were orally inoculated with cell culture adapted PEDV (PEDV EU) four weeks before farrowing to induce MDA-positive piglets (group A1). Another two sows (ear tags 4423 and 6150) were treated the same way orally with German field material (organ suspension and fecal homogenate; DE) containing PEDV to also obtain MDA-positive piglets (group B1). The remaining four sows stayed untreated to obtain MDA-negative piglets (group A2: ear tags 4343 and 4454; group B2: ear tags 1866 and 4365).

All piglets born alive were individually ear tagged prior to challenge inoculation. Piglets born to sows of groups A1 (9 piglets of sow 4249 and 12 piglets of sow 1871) and A2 (7 piglets of sow 4454 and 7 piglets of sow 4343) were orally challenged with PEDV EU at an age of three to six days whereas piglets born to sows of groups B1 (7 piglets of sow 6150 and 5 piglets of sow 4423) and B2 (8 piglets of sow 4365 and 8 piglets of sow 1866) received the German field material (DE).

During the whole trial, daily rectal swabs (COPAN plain cotton swabs without medium) were taken of all animals for real-time reverse transcription polymerase chain reaction (RT-qPCR) analyses. Samples of days 0 to 7 post inoculation (pi) as well as days 10 pi and 13 pi were tested. Moreover, samples were tested from day 21 pi till the end of the trial.

Additional rectal swabs were taken of four randomly chosen piglets of each sow prior to inoculation and two days afterwards for bacteriological examination. Moreover, clinical signs indicative for PED were recorded using a standardized score system (see Table 2). Blood samples were taken at the day of inoculation and the day of slaughter or euthanasia. Additional blood and colostrum samples were collected from all sows during farrowing. Furthermore, milk samples were collected from all but two sows at the day of slaughter (samples missing from sow 4249 which died from septicemia, and sow 1871 which milk production had already ceased). At the end of the trial, all remaining piglets were euthanized and seven sows were slaughtered (electro-stunning and subsequent exsanguination). All animals were necropsied and samples of the small intestine were taken.

### Viruses and field virus suspensions

For inoculation of animals of groups A1 and A2, cell culture adapted PEDV was provided by the Boehringer Ingelheim Veterinary Research Center (PEDV EU). This cell culture isolate was obtained from a PED outbreak in Northern Germany with high mortality rates in suckling piglets. It is sharing over 99% nucleotide identity with the U.S. strain OH851 and recent Central European PEDV strains. The initial isolate was obtained using the following (standard) protocol: 5 × 10^5^ Vero cells were seeded into 6-wells plates. After 24 h of incubation at 37°C, confluent monolayers were obtained and the cell culture medium was removed. All wells were washed three times with 3 ml phosphate-buffered saline (PBS). Thereafter, cells were inoculated with 200 µl of filtrated (0.22 µm) homogenate from piglet guts. After the addition of 2 ml of PEDV media containing 15 µg/ml trypsin, cultures were incubated for 24–48 h at 37°C. The cultures were checked daily for the presence of characteristic fusion formation, and positive materials were further passaged. For the purpose of our study, the titer was defined by end point titration and recorded as tissue culture infection dose_50_ (TCID_50_)/ml. The stock titer was 3.16 × 10^5^ TCID_50_/ml (RT-qPCR: cq value 15). Each sow of group A1 received 6 ml of this stock diluted with 14 ml PBS. The solution was orally fed using a 20 ml syringe. Piglets of groups A1 and A2 were also orally inoculated. In this case, each piglet received 2 ml of a 1:10 diluted viral stock (titer 3.16 × 10^4^ TCID_50_/ml) using 2 ml syringes.

To inoculate animals of groups B1 and B2, PEDV-PCR-positive field material (DE) from recent clinical cases in South-Western Germany was used. In these cases mainly fattening pigs were affected by rather mild clinical signs. The material was a pool of different fecal samples and intestines. To obtain the inoculum, intestines were homogenized with sterile sea sand using mortar and pestle. Faecal samples were mixed and also homogenized. Each sow of group B1 received 20 ml of the organ suspension (RT-qPCR: cq value 23) and 20 ml of the faecal homogenate (RT-qPCR: cq value 15) orally using 20 ml syringes. All piglets of groups B1 and B2 received a faecal homogenate obtained from the sows of group B1 at 5 dpi and 6 dpi (RT-qPCR: cq value 19). The piglets were inoculated orally with 2 ml of this faecal homogenate using 2 ml syringes.

### Sample preparation and nucleic acid extraction

Rectal swabs were submerged in 1 ml Dulbecco’s Modified Eagle Medium with standard antibiotics and antimycotics (Antibiotic-Antimycotic 100×, Gibco) and incubated for 1 hour at room temperature. Viral RNA was extracted using either the manual QIAmp ViralRNA Mini Kit (Qiagen) or the NucleoMagVet-Kit (Macherey-Nagel) in combination with the KingFisher extraction platform (Thermo Scientific). The RNA was stored at −20 °C until further use.

Blood samples were centrifuged at 2000 × g for 20 minutes at room temperature to obtain serum. The resulting serum was aliquoted and stored at −20 °C. Colostrum and milk samples were aliquoted and stored at −20 °C until further use.

### Virus detection

To detect PEDV genome in fecal swabs, a RT-qPCR system targeting the S-gene of PEDV was used as described previously^33^. Cq values above 40 were considered negative and the amount of PEDV genome copies was calculated by using a standard curve.

In addition, the inoculum for sows and piglets was tested for porcine circovirus 2, porcine enteroviruses (including enteroviruses, teschoviruses, and sapeloviruses), rotavirus A, transmissible gastroenteritis virus, and porcine delta coronavirus using specific in-house RT-PCR assays (primer sequences and reaction conditions are available from the authors upon request).

Swabs of animals which showed central nervous signs that could have been indicative for infection with teschoviruses were also tested in the PEV PCR assay (concerns the litters of sows 4423 and 6150, group B1).

### Plots and statistics

Creation of different plots and charts was performed using SigmaPlot for Windows version 11.0 (Systat Software). Shapiro-Wilk test was used for normality testing and a Mann-Whitney rank sum test was conducted as implemented in the software package. Statistical significance (p <= 0.05 was considered significant) was tested using SigmaPlot software.

### Veterinary treatments unrelated to PED

Six sows (all except animals 4249 and 1871) received cloprostenol (Estrumate, Intervet) to induce farrowing. Moreover, all sows were treated with antibiotics (Riketron N, aniMedica) and anti-inflammatory drugs (Metacam, Boehringer) for a mild Metritis-Mastitis-Agalactia-Syndrome over 3 days. Piglets of sow No. 6150 (group B1) received Baytril orally over 4 days because of mild enteritis prior to inoculation. Where needed, piglets received anti-inflammatory drugs (Metacam, Boehringer) for lameness (arthritis, panaritia).

### Antibody detection

Three commercial indirect ELISA (Swinecheck PED indirect, BIOVET, GC Kerkrade, the Netherlands; INgezim PEDV, INGENASA, Madrid, Spain; ID Screen PEDV indirect, Grabels, France) were performed with all sera according to the producer’s manual. Colostrum and milk samples were tested in the same manner after initial validation. While ELISA kits of BIOVET and IDVet are using recombinant nucleoprotein, the INGENASA ELISA plates are coated with recombinant spike protein.

In cases of ambiguous results, the respective samples were tested in indirect immunofluorescence assays using commercial PEDV FA Substrate Slides (VMRD, Pullman, Washington) following the manufacturer’s instructions. The slides were screened for specific fluorescence with a standard fluorescence microscope (Zeiss Axio Vert.A1, Oberkochen, Germany).

In addition, colostrum and milk samples were tested for PEDV specific IgA antibodies using an in-house indirect ELISA (assay specifications are available from the authors upon request).

### Data availability statement

The datasets generated and analysed during the current study are available from the corresponding author on reasonable request.

## Electronic supplementary material

Clinical Score values of sows and IgA in colostrum and milk samples
