# Supplementary material for: Virulence of current German PEDV strains in suckling pigs and investigation of protective effects of maternally derived antibodies
Source: Sci Rep. 2017 Sep 7;7:10825. doi: 10.1038/s41598-017-11160-w (PMC5589859; doi:10.1038/s41598-017-11160-w)
Supplement: Supplementary file 1 — Clinical Score values of sows and IgA in colostrum and milk samples [file 41598_2017_11160_MOESM1_ESM.pdf]

Virulence of current German PEDV strains in suckling pigs and investigation of protective effects of maternally derived antibodies

Leidenberger, S.[1]; Schröder, Ch.[1]; Zani, L.[1]; Auste, A.[1]; Pinette, M.[2], Ambagala, A.[2]; Nikolin, V.[3]; de Smit, H.[3]; Beer, M.[1] and Blome, S.[1*]

[1] *Institute of Diagnostic Virology, Friedrich-Loeffler-Institut, Suedufer 10, Greifswald - Insel Riems, 17493, Germany*

[2] *Canadian Food inspection Agency, National Centre for Foreign Animal Disease, Winnipeg, R3E 3M4, Canada*

[3] *Boehringer Ingelheim Veterinary Research Center, Hannover, 30559, Germany*

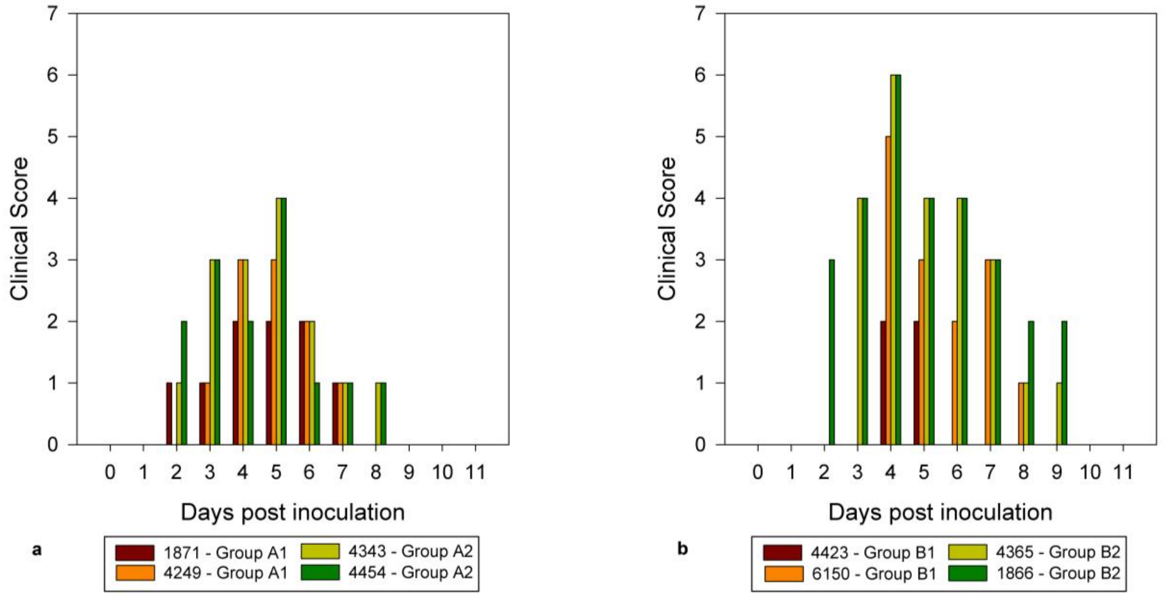

***Supplementary Fig. F1:* Clinical Score values of sows:** chart a: sows infected with cell culture strain of PEDV (PEDV-EU), chart b: sows infected with German field material containing PEDV; sows belonging to group A1 and B1 were orally infected prior to farrowing and sows belonging to group A2 and B2 got infected through PEDV shedding of their respective piglets.

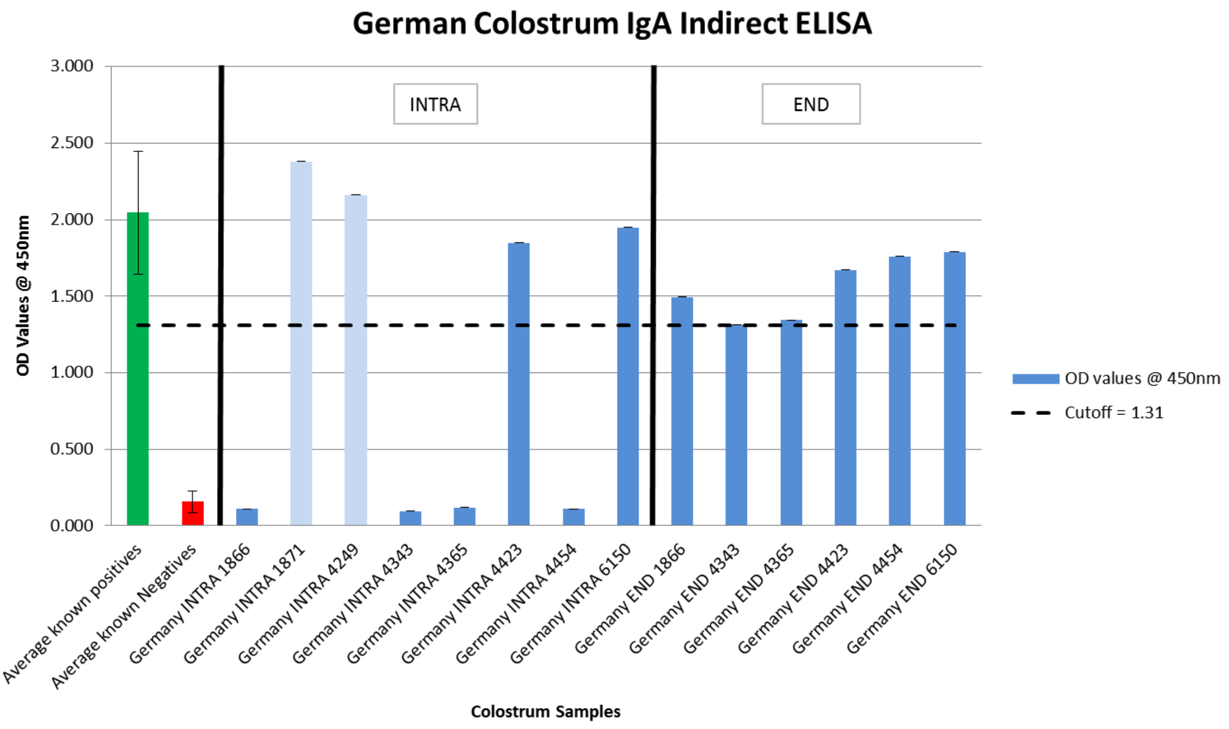

Supplementary Fig. F2: **IgA in colostrum and milk samples:** Amount of IgA antibodies in colostrum taken intra partum and milk samples at the end of the study; sows 1871 and 4249 belong to group A1, sows 4343 and 4454 belong to group A2, sows 6150 and 4423 belong to group B1 and sows 1866 and 4365 belong to group B2
